# Neural Network Self-Tuning Control for a Piezoelectric Actuator

**DOI:** 10.3390/s20123342

**Published:** 2020-06-12

**Authors:** Wenjun Li, Chen Zhang, Wei Gao, Miaolei Zhou

**Affiliations:** 1 College of Transportation, Jilin University, Changchun 130022, China; liwj@jlu.edu.cn; 2 College of Communication Engineering, Jilin University, Changchun 130022, China; zhangchen19@mails.jlu.edu.cn (C.Z.); gaow@jlu.edu.cn (W.G.)

**Keywords:** hysteresis, piezoelectric actuator, neural network, self-tuning

## Abstract

Piezoelectric actuators (PEA) have been widely used in the ultra-precision manufacturing fields. However, the hysteresis nonlinearity between the input voltage and the output displacement, which possesses the properties of rate dependency and multivalued mapping, seriously impedes the positioning accuracy of the PEA. This paper investigates a control methodology without the hysteresis model for PEA actuated nanopositioning systems, in which the inherent drawback generated by the hysteresis nonlinearity aggregates the control accuracy of the PEA. To address this problem, a neural network self-tuning control approach is proposed to realize the high accuracy tracking with respect to the system uncertainties and hysteresis nonlinearity of the PEA. First, the PEA is described as a nonlinear equation with two variables, which are unknown. Then, using the capabilities of super approximation and adaptive parameter adjustment, the neural network identifiers are used to approximate the two unknown variables automatically updated without any off-line identification, respectively. To verify the validity and effectiveness of the proposed control methodology, a series of experiments is executed on a commercial PEA product. The experimental results illustrate that the established neural network self-tuning control method is efficient in damping the hysteresis nonlinearity and enhancing the trajectory tracking property.

## 1. Introduction

**Figure 1:**
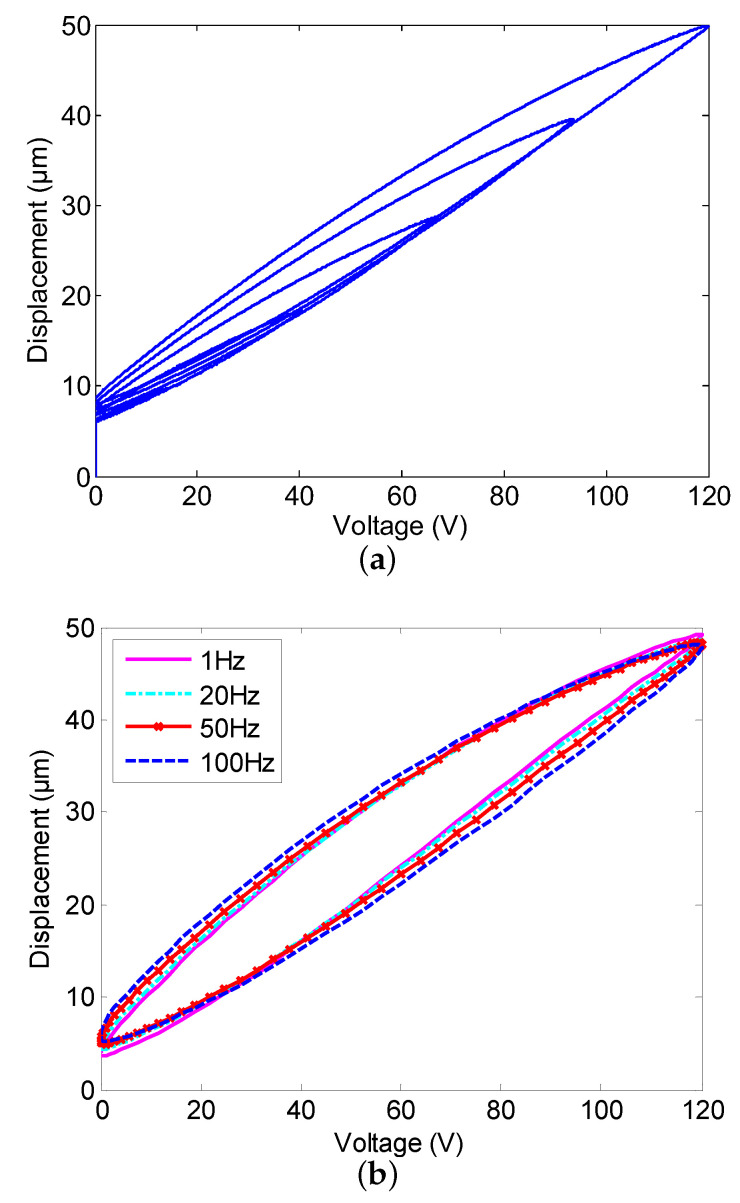
Actual input-output hysteresis curves of a piezoelectric actuator (PEA). (**a**) multivalued mapping behavior; (**b**) rate-dependent behavior.

A piezoelectric actuator (PEA) is a transducer that converts electrical energy into a mechanical displacement or stress based on a piezoelectric effect, or vice versa [1,2]. Compared with traditional electromechanical systems, the PEA actuated nanopositioning system has primary advantages [3,4,5] including the large mass ratio, sub-nanometer resolution, high positioning accuracy, and fast response characteristic; hence, it is extensively applied in atomic force microscope [6], ultra-precision mechanical control [7], biological manipulation [8], and other related fields. Nevertheless, the dominant challenge of the PEA derives from the hysteresis nonlinearity, which impedes the nanopositioning systems from obtaining the fine property notably [9]. Hysteresis nonlinearity is the non-smooth nonlinear behavior between the input voltage and the output displacement of the PEA [10,11]. Hysteresis nonlinearity is multivalued and nonlocal memoryless, which brings on the multivalued mapping behavior [12,13], as shown in Figure 1a. As the amplitude of the input signal increases, the input-output curves of the PEA will generate different hysteresis loops. Furthermore, the maximum value of the displacement changes from 40 μm to 120 μm, and this phenomenon indicates that the output displacement of the PEA is not only dependent on the current value of the input voltage, but also is related to the historical value. Beyond that, the work in [14] declared that the hysteresis nonlinearity is a rate-dependent behavior likewise, as shown in Figure 1b. The shape of the hysteresis loops becomes wider gradually as the frequency of the input voltage is increased from 1 Hz to 100 Hz, which means that the input frequency of the driven signal is the inevitable factor to effect the output displacement. In addition, along with the growing of the input voltage frequency, the tracking errors caused by the hysteresis nonlinearity increase as well. To meet such a challenge, some control schemes have been proposed for the PEA during the past decade. The feedforward control approach is most widely used for compensating the hysteresis nonlinearity, structuring a controller by placing in a feedforward loop as a compensator with various models. These models include the Preisach model [15], the Prandtl–Ishlinskii model [16,17,18], the Krasnoselskii–Pokrovskii model [19], the Bouc–Wen model [20,21], the Duhem model [22,23], the Dahl model [24], and the neural network model [25,26] to suppress the undesirable behaviors. It is pointed out that the precision of open-loop control is affected by modeling errors concerning these hysteresis models; furthermore, open-loop control cannot suppress the influence of external interference.

To overcome this problem, feedback control [27,28,29,30] is developed for high-precision control of the PEA. Al-Ghanimi et al. designed a fast non-singular terminal sliding mode controller based on perturbation estimation [27], and experimental results verified that this controller could track a reference signal precisely in the case of modeling error, external perturbation, and the system parameters’ uncertainty. In [28], a composite control approach was developed for a piezo-actuated stage to track the desired reference trajectory accurately. An inverse multiplicative structure based feedforward hysteresis compensator was used to eliminate the hysteresis nonlinearity. Then, the linear model predictive control, which had a simple and clear solution, was adopted to achieve the precise tracking performance. On the basis of constructing a Bouc–Wen model to characterize the hysteresis behavior of the PEA, Chen et al. introduced an internal model based controller, which guaranteed that the desired reference signal could be tracked under any initial conditions of the close-loop system [29]. The hysteresis nonlinearity of the PEA is either directly regarded as a disturbance or represented by the hysteresis models. By using the hysteresis model to describe the hysteresis nonlinearity, the inverse compensator with modified the Prandtl–Ishlinskii model [30] was designed to compensate for the hysteresis characteristic of a PEA, and a disturbance observer was used to compensate for the lumped disturbance and system uncertainties, then a tracking controller was adopted to further decrease errors. An adaptive tracking controller [31] was designed using the LuGre model and achieved fine performance in displacement tracking. In [32], sliding mode control with a sigmoid function was developed for the tracking control of the piezo-actuated stage, in which the Bouc–Wen model was adopted to describe the hysteresis nonlinearity. Similar to the control scheme in [32], the repetitive controller [33] was developed to improve the tracking capability of the piezo-actuated stage with the Prandtl–Ishlinskii model. By treating the hysteresis nonlinearity as a nonlinear bounded disturbance, an approximate hysteresis model that combined a variable gain and a variable time-delay term was proposed, and on the basis of this model, a Smith predictor based H${}_{\infty }$ controller [34] was designed to implement high-precision motion tracking control of a piezo-actuated stage. Meanwhile, an integrated PID based sliding mode controller [35] was presented to enhance control performance with a novel PID based sliding mode observer for the unknown disturbances. The designed observer relaxed the traditional matching conditions when establishing the unknown-input observers. Experimental results showed that the presented control method could estimate the PEA state precisely and attain an excellent tracking effect.

Most control methods are model based to compensate for the hysteresis nonlinearity. However, the uncertainties of the system models and the complex hysteresis nonlinear behavior make it difficult to describe adequately. Neural networks have powerful capabilities such as input-output mapping, function approximation, and adaptability [36,37,38,39], which can approximate the system dynamics and the hysteresis nonlinear behavior. In [40], a feedforward neural network controller was proposed to improve the tracking precision of the PEA, and the bat search algorithm was adopted to decrease the computational burden. Simulation results indicated that this developed method could considerably improve the steady-state error and settling time. Lin et al. designed an adaptive wavelet neural network controller with hysteresis estimation for a piezo-positioning mechanism in [41]. According to the theoretical analysis and experimental results, the asymptotic stability of the proposed controller was guaranteed. Furthermore, the robustness and the tracking performance were greatly improved. In [42], a control strategy of utilizing the neural network as the system feedback controller was proposed, and by conducting the simulations with the collected data, the effectiveness of the developed approach was validated and the positioning precision of the PEA could be improved greatly.

Self-tuning control is a kind of adaptive control technique, for which the parameters of the object are estimated online via the identifier, and then, these parameters can be adjusted automatically by the controller [43,44]. When applying the traditional self-tuning control, the object is generally described by a linear or linearized model to achieve the control effect. However, for a complex nonlinear system with the hysteresis behavior, such as the PEA, it is a challenging task to design the self-tuning control. The self-tuning control has the merits of accommodating the system uncertainty and suppressing random disturbances effectively. In [45], a neural network based self-tuning PID control was utilized to deal with the problem of the fixed gain existing in PID control, and this control approach was implemented on an underwater remotely-operated vehicle in real-time. Experimental results verified the effectiveness of the proposed method. The work in [46] introduced a self-tuning proportional double derivative-like neural network controller for an unmanned aerial vehicle in the presence of parametric uncertainties and unknown disturbances. Various kinds of self-tuning controllers have been successfully applied in different nonlinear systems and have shown excellent control performance. Some scholars have investigated the tracking problem of the PEA via self-tuning neuro-PID control [47] and fuzzy-reasoning based self-tuning PID control methods [48]. However, the positioning issue in the presence of the hysteresis nonlinearity of the PEA has not been fully discussed by using the neural network based self-tuning control approach. Hence, in this paper, the self-tuning control is combined with the neural network to construct the neural network self-tuning controller for the PEA. Unlike the model based controllers, which need to obtain an accurate hysteresis model in advance, the PEA is described by the nonlinear function expression with two unknown variables, and two neural networks are applied as the identifiers to approximate the unknown variables of the PEA in online self-tuning control, so that the proposed controller can track the desired reference trajectory with high precision. Experiments are made on a commercial PEA product, and the results illustrate the control performance of the PEA in trajectory tracking.

The rest of this paper is organized as follows. In Section 2, the principle of the neural network self-tuning control algorithm is introduced, and the neural network identifiers are designed to achieve the high precision tracking for the PEA in the presence of hysteresis nonlinearity. Then, the experimental validations under the sinusoidal reference trajectories with various frequencies and the mixed triangular reference trajectory are conducted in Section 3. Finally, this paper is concluded in Section 4.

## 2. Controller Design

In practical operation, such as the micro-positioning system, the hysteresis nonlinearity that widely exists in the PEA can seriously deteriorate the systems performance and even leads to the oscillation and instability of the actual system. Therefore, the objective of our developed controller is to eliminate the hysteresis nonlinearity and achieve the high-precision tracking effect, so as to facilitate the further application of the PEA as a crucial driving mechanism. To realize this aim, the neural network self-tuning control scheme is developed in this paper.

### 2.1. Design Philosophy

The neural network has distinguished abilities of nonlinearity approximation and adaptiveness. Taking the shortcomings of the conventional self-tuning control into account, the neural network self-tuning control algorithm [49] is adopted in this paper to achieve the tracking precision of the PEA.

The PEA is described as the nonlinear equation as follows:

(1) $$y\left(k+1\right)=g\left[y\left(k\right)\right]+f\left[y\left(k\right)\right]u\left(k\right)$$

where $y\left(k\right)$ is the hysteresis output, $u\left(k\right)$ is the input voltage, and $g\left[y\left(k\right)\right]$ and $f\left[y\left(k\right)\right]$ are nonlinear functions. *k* denotes the $k^{\mathrm{th}}$ training time.

When $g\left[y\left(k\right)\right]$ and $f\left[y\left(k\right)\right]$ are exactly known, according to Equation (1), the control law can be derived as follows:

(2) $$u\left(k\right)=\frac{y\left(k+1\right)-g\left[y\left(k\right)\right]}{f\left[y\left(k\right)\right]}$$

The output $y\left(k+1\right)$ of the PEA can exactly track the desired reference signal $y_{d}\left(k+1\right)$, namely:

(3) $$y\left(k+1\right)=y_{d}\left(k+1\right)$$

Substituting Equation (3) into Equation (2), the control law Equation (2) is restated as follows:

(4) $$u\left(k\right)=\frac{y_{d}\left(k+1\right)-g\left[y\left(k\right)\right]}{f\left[y\left(k\right)\right]}$$

However, it is tough to describe the uncertainties of the PEA and its complex hysteresis nonlinear behavior sufficiently. Therefore, $g\left[y\left(k\right)\right]$ and $f\left[y\left(k\right)\right]$ are commonly unknown. To solve this problem, two neural network identifiers Ng and Nf are adopted to approximate the nonlinear functions $g\left[y\left(k\right)\right]$ and $f\left[y\left(k\right)\right]$, respectively. Then, the control law in Equation (4) is rewritten as follows:

(5) $$u\left(k\right)=\frac{y_{d}\left(k+1\right)-Ng\left[y\left(k\right)\right]}{Nf\left[y\left(k\right)\right]}$$

where the estimated values of $g\left[y\left(k\right)\right]$ and $f\left[y\left(k\right)\right]$ are represented by $Ng\left[y\left(k\right)\right]$ and $Nf\left[y\left(k\right)\right]$, respectively.

**Figure 2:**
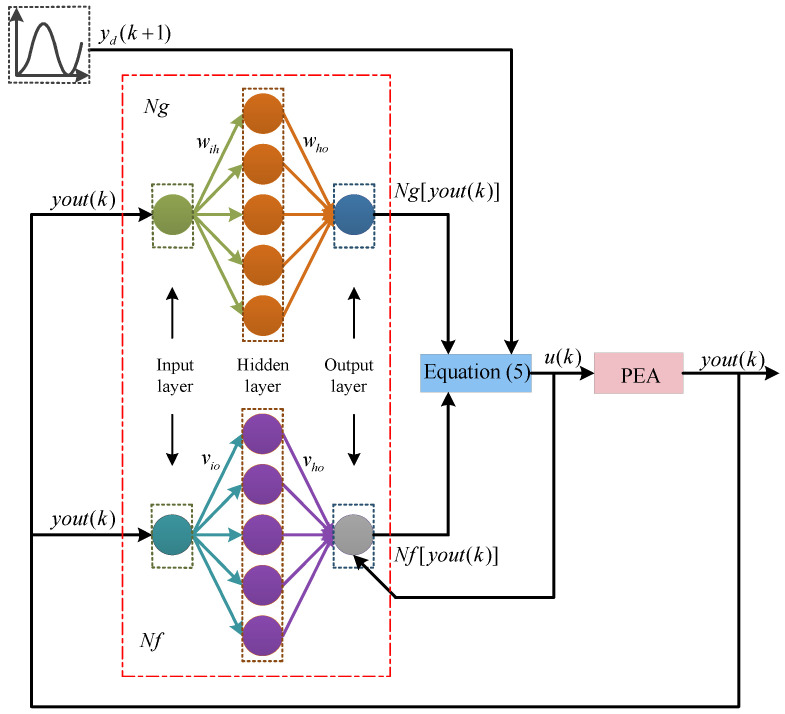
The block diagram of the proposed neural network self-tuning control scheme for PEA.

The control goal is that the hysteresis output $y\left(k+1\right)$ can track a given signal $y_{d}\left(k+1\right)$ exactly. The actual hysteresis output $y\left(k\right)$ is fed into the input of back-propagation neural network identifiers Ng and Nf, which have the ability to “learn” system features based on nonlinear mapping. Both the input layer and output layer of the neural network identifiers adopt one neuron, and the variables of the neuron in the hidden layer are five. Then, the estimated control variable $u\left(k\right)$ in Equation (5) is adopted to control the PEA. The block diagram of the proposed neural network self-tuning control scheme for the PEA is depicted in Figure 2.

The estimated hysteresis output $\tilde{y}\left(k+1\right)$ of the PEA is expressed as follows:

(6) $$\begin{array}{cc} \tilde{y}\left(k+1\right)= & Ng\left[y\left(k\right)\right]+Nf\left[y\left(k\right)\right]u\left(k\right)\\ = & Ng\left[y\left(k\right);W\left(k\right)\right]+Nf\left[y\left(k\right);V\left(k\right)\right]u\left(k\right) \end{array}$$

(7) $$W\left(k\right)=\left[w_{0},w_{1}\left(k\right),w_{2}\left(k\right),w_{3}\left(k\right),w_{4}\left(k\right),w_{5}\left(k\right)\right]$$

(8) $$V\left(k\right)=\left[v_{0},v_{1}\left(k\right),v_{2}\left(k\right),v_{3}\left(k\right),v_{4}\left(k\right),v_{5}\left(k\right)\right]$$

where $W\left(k\right)$ and $V\left(k\right)$ represent the weights of the two neural network identifiers Ng and Nf, respectively. The sigmoid Equation (9) is selected as the activation function of the hidden layer in neural network identifier Ng. The hyperbolic tangent Equation (10) is selected as the activation function of the hidden layer in neural network identifier Nf.

(9) $$f_{Ng}\left(x\right)=\frac{1-e^{-x}}{1+e^{-x}}$$

(10) $$f_{Nf}\left(x\right)=\frac{e^{x}-e^{-x}}{e^{x}+e^{-x}}$$

Equation (5) is substituted into Equation (1), and the hysteresis output of the PEA is described as follows:

(11) $$\begin{array}{cc} y\left(k+1\right)= & f\left[y\left(k\right)\right]\frac{y_{d}\left(k+1\right)-Ng\left[y\left(k\right)\right]}{Nf\left[y\left(k\right)\right]}+g\left[y\left(k\right)\right] \end{array}$$

It is obvious that when the following Equations (12) and (13) are satisfied, the actual hysteresis output $y\left(k+1\right)$ can be infinitely close to the desired displacement output $y_{d}\left(k+1\right)$, namely $y\left(k+1\right)$ is equivalent to $y_{d}\left(k+1\right)$.

(12) $$Ng\left[y\left(k\right);W\left(k\right)\right]\to g\left[y\left(k\right)\right]$$

(13) $$Nf\left[y\left(k\right);V\left(k\right)\right]\to f\left[y\left(k\right)\right]$$

### 2.2. Neural Network Identifiers Ng and Nf

In the overall control algorithm, the input vector of input layer for neural network identifiers Ng and Nf is $y\left(k\right)$. $W\left(k\right)$ and $v\left(k\right)$ are the weight vectors for training in neural network identifiers Ng and Nf, respectively. Then, the optimal $W\left(k\right)$ and $v\left(k\right)$ can minimize the mean squared error formula, that is:

(14) $$\begin{array}{c} E\left(k\right)=\frac{1}{2}{\left[y_{d}\left(k+1\right)-y\left(k+1\right)\right]}^{2}=\frac{1}{2}e^{2}\left(k+1\right) \end{array}$$

where e(k+1) is the error between $y_{d}\left(k+1\right)$ and y(k+1).

Then, updated weight Δw in neural network identifier Ng and updated weight Δv in neural network identifier Nf are adjusted such that $E\left(k\right)$ can be reduced. It can be verified as follows:

(15) $$\begin{array}{c} \Delta w\left(k\right)=\eta_{w}\frac{\partial E\left(k\right)}{\partial w\left(k\right)}=\eta_{w}\frac{\partial E\left(k\right)}{\partial y\left(k\right)}\frac{\partial y\left(k\right)}{\partial w\left(k\right)} \end{array}$$

(16) $$\begin{array}{c} \Delta v\left(k\right)=\eta_{v}\frac{\partial E\left(k\right)}{\partial v\left(k\right)}=\eta_{v}\frac{\partial E\left(k\right)}{\partial y\left(k\right)}\frac{\partial y\left(k\right)}{\partial v\left(k\right)} \end{array}$$

where $\eta_{w}$ and $\eta_{v}$ are the learning rate, which compensate for the weight adjustment error in neural network identifiers Ng and Nf, respectively.

Equations (11) and (14) are substituted into Equation (15), and the algorithm of weight adjustment in the neural network identifier Ng is derived as follows:

(17) $$\begin{array}{cc} \Delta w(k)=\; & \eta_{w}\frac{\partial \left(\frac{1}{2}e^{2}\left(k+1\right)\right)}{\partial y\left(k\right)}\frac{\partial y\left(k\right)}{\partial w\left(k\right)}\\ = & \frac{\partial \left[g\left[y\left(k\right)\right]+f\left[y\left(k\right)\right]\frac{y_{d}\left(k+1\right)-Ng\left[y\left(k\right);W\left(k\right)\right]}{Nf\left[y\left(k\right);V\left(k\right)\right]}\right]}{\partial y\left(k\right)}\left(-\eta_{w}\right)\frac{\partial y\left(k\right)}{\partial w\left(k\right)}e\left(k+1\right)\\ = & \eta_{w}\frac{f\left[y\left(k\right)\right]}{Nf\left[y\left(k\right);V\left(k\right)\right]}\frac{\partial Ng\left[y\left(k\right);W\left(k\right)\right]}{\partial w\left(k\right)}e\left(k+1\right) \end{array}$$

In the neural network identifier Ng, $w_{ih}$ represents the weight matrix between the input and the hidden layer, and $w_{ho}$ represents the weight matrix between the hidden and the output layer.

The process of weight adjustment between the hidden and the output layer in the neural network identifier Ng is expressed as follows:

(18) $$\begin{array}{cc} \frac{\partial Ng\left[y\left(k\right);W\left(k\right)\right]}{\partial w_{ho}}= & \frac{\partial Ng\left[y\left(k\right);W\left(k\right)\right]}{\partial Net1_{o}^{i}}\frac{\partial Net1_{o}^{i}}{\partial w_{ho}}\\ = & Ng\left[y\left(k\right);W\left(k\right)\right]OH1_{h}\left(1-Ng\left[y\left(k\right);W\left(k\right)\right]\right) \end{array}$$

where $Net1_{o}^{i}$ represents the input of the output layer node *o* in the neural network identifier Ng and $OH1_{h}$ represents the output vector of the hidden layer. Equation (18) is substituted into Equation (17), then we can obtain:

(19) $$\begin{array}{cc} \Delta w_{ho}= & \eta_{w}\frac{f[y\left(k\right)]}{Nf[y\left(k\right);V\left(k\right)]}\frac{\partial Ng[y\left(k\right);W\left(k\right)]}{\partial w_{ho}\left(k\right)}e\left(k+1\right)\\ = & \eta_{w}\frac{f[y\left(k\right)]}{Nf[y\left(k\right);V\left(k\right)]}Ng\left[y\left(k\right);W\left(k\right)\right]\left(1-Ng[y\left(k\right);W\left(k\right)]\right)OH1_{h}e\left(k+1\right) \end{array}$$

Although $f\left[y\left(k\right)\right]$ is unknown, it can be assumed that its notation is known. Beyond that, the positive and negative of $f\left[y\left(k\right)\right]$ determine the convergence direction in the weight adjustment algorithm. Therefore, $f\left[y\left(k\right)\right]$ is replaced by $\mathrm{sgn}\{f\left[y\left(k\right)\right]\}$ before (19) is used in the next update criterion.

(20) $$\begin{array}{cc} w{}_{ho}\left(k\right)= & w{}_{ho}\left(k-1\right)+\Delta w{}_{ho}\left(k-1\right)+\alpha_{1}\left(w{}_{ho}\left(k-1\right)-w{}_{ho}\left(k-2\right)\right)\\ = & w{}_{ho}\left(k-1\right)+\eta_{w}\frac{\mathrm{sgn}\{f\left[y\left(k\right)\right]\}}{Nf[y\left(k\right);V\left(k\right)]}e\left(k+1\right)Ng\left[y\left(k\right);W\left(k\right)\right]\left(1-Ng\left[y\left(k\right);W\left(k\right)\right]\right)OH1_{h}\\ \; & +\alpha_{1}\left(w{}_{ho}\left(k-1\right)-w{}_{ho}\left(k-2\right)\right) \end{array}$$

where $\alpha_{1}$ is a constant.

The process of weight adjustment between the input and the hidden layer in the neural network identifier Ng is described as follows:

(21) $$\begin{array}{cc} \frac{\partial Ng\left[y\left(k\right);W\left(k\right)\right]}{\partial w_{ih}}= & \frac{\partial Ng\left[y\left(k\right);W\left(k\right)\right]}{\partial OH1_{h}}\frac{\partial OH1_{h}}{\partial Net1_{h}^{i}}\frac{\partial Net1_{h}^{i}}{\partial w_{ih}}\\ = & \frac{\partial Ng\left[y\left(k\right);W\left(k\right)\right]}{\partial OH1_{h}}OH1_{h}\left(1-OH1_{h}\right)I_{1} \end{array}$$

where $Net1_{h}^{i}$ represents the input of the hidden layer node *h* in the neural network identifier Ng and $I_{1}$ is the input vector of the hidden layer.

(22) $$\begin{array}{cc} \frac{\partial Ng\left[y\left(k\right);W\left(k\right)\right]}{\partial OH1_{h}}= & \frac{\partial Ng\left[y\left(k\right);W\left(k\right)\right]}{\partial Net1_{o}^{i}}\frac{\partial Net1_{o}^{i}}{\partial OH1_{h}}\\ = & Ng\left[y\left(k\right);W\left(k\right)\right]\left(1-Ng\left[y\left(k\right);W\left(k\right)\right]\right)w_{ho} \end{array}$$

Equations (21) and (22) are substituted into Equation (17), and we can obtain:

(23) $$\begin{array}{cc} \Delta w_{ih}= & \eta_{w}\frac{f[y\left(k\right)]}{Nf[y\left(k\right);V\left(k\right)]}\frac{\partial Ng[y\left(k\right);W\left(k\right)]}{\partial w_{ih}\left(k\right)}e\left(k+1\right)\\ = & Ng\left[y\left(k\right);W\left(k\right)\right]\left(1-Ng[y\left(k\right);W\left(k\right)]\right)e\left(k+1\right)\eta_{w}\frac{f[y\left(k\right)]}{Nf[y\left(k\right);V\left(k\right)]}OH1_{h}\left(1-OH1_{h}\right)I_{1}w_{ho} \end{array}$$

In the same way, $f\left[y\left(k\right)\right]$ is replaced by $\mathrm{sgn}\{f\left[y\left(k\right)\right]\}$, and the learning rate $\eta_{w}$ is used to compensate for the weight adjustment error caused by approximate substitution.

(24) $$\begin{array}{cc} w{}_{ih}\left(k\right)= & w{}_{ih}\left(k-1\right)+\Delta w{}_{ih}\left(k-1\right)+\alpha_{1}\left(w{}_{ih}\left(k-1\right)-w{}_{ih}\left(k-2\right)\right)\\ = & \eta_{w}\frac{\mathrm{sgn}\{f\left[y\left(k\right)\right]\}}{Nf\left[y\left(k\right);V\left(k\right)\right]}Ng\left[y\left(k\right);W\left(k\right)\right]\left(1-Ng[y\left(k\right);W\left(k\right)]\right)OH1_{h}\left(1-OH1_{h}\right)I_{1}e\left(k+1\right)w_{ho}\\ \; & +w{}_{ih}\left(k-1\right)+\alpha_{1}\left(w{}_{ih}\left(k-1\right)-w{}_{ih}\left(k-2\right)\right) \end{array}$$

Similar to the progress solving for $w_{ho}$ and $w_{ih}$ of the neural network identifier Ng, we can obtain the weight matrix $v_{ho}$ between the hidden and the output layer of the neural network identifier Nf and the weight adjustment $v_{ih}$ between the input and the hidden layer of the neural network identifier Nf.

(25) $$\begin{array}{cc} v{}_{ho}\left(k\right)= & v{}_{ho}\left(k-1\right)+\eta_{v}\mathrm{sgn}\left\{f\left[y\left(k\right)\right]\right\}\left(1-Nf[y\left(k\right);V\left(k\right)]\right)OH2_{h}e\left(k+1\right)u\left(k\right)\\ \; & +\alpha_{2}\left(v{}_{ho}\left(k-1\right)-v{}_{ho}\left(k-2\right)\right) \end{array}$$

(26) $$\begin{array}{cc} v{}_{ih}\left(k\right)= & v{}_{ih}\left(k-1\right)+\eta_{v}\mathrm{sgn}\left\{f\left[y\left(k\right)\right]\right\}e\left(k+1\right)u\left(k\right)v_{ho}\left(1-Nf\left[y\left(k\right);V\left(k\right)\right]\right)OH2_{h}\left(1-OH2_{h}\right)I_{2}\\ \; & +\alpha_{2}\left(v{}_{ih}\left(k-1\right)-v{}_{ih}\left(k-2\right)\right) \end{array}$$

where $OH2_{h}$ represents the output vector of the hidden layer in the neural network identifier Nf and $\alpha_{2}$ is a constant.

## 3. Experimental Study

**Figure 3:**
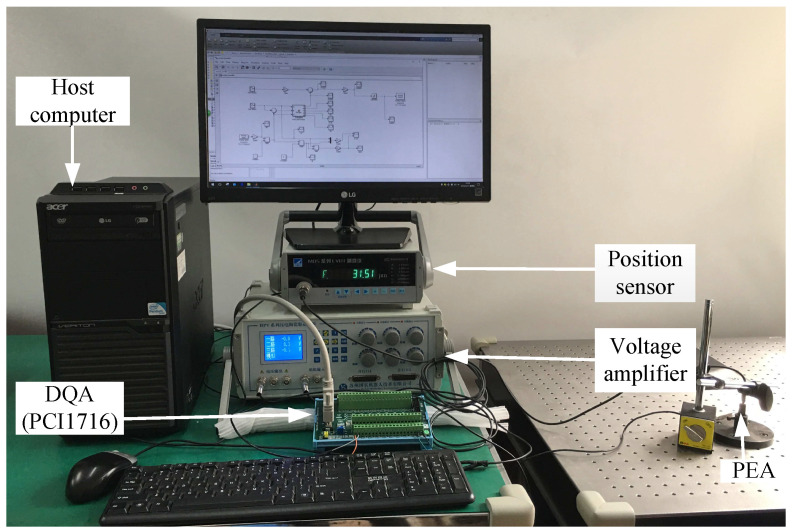
Picture of the experimental setup.

To confirm the efficiency of the proposed neural network self-tuning control method, plenty of experiments were executed on a commercial PEA product, in which the trajectory tracking performances were certified through a PC based control system. The experimental system, shown in Figure 3, was composed of a data acquisition (DAQ) interface (PCI-1710, Advantech, Taiwan, China), a voltage amplifier (HPV-3C, SuZhou Micro Automation Technology Co., Ltd, Suzhou China), a PEA (PZT 150/7×7/50, SuZhou Micro Automation Technology Co., Ltd, Suzhou China), a position sensor (MDSL-0500M6-1A, SuZhou Micro Automation Technology Co., Ltd, Suzhou China), and a host computer. The specifications of the considered PEA product are presented in Table 1. The input voltage for the PEA was sent to actuate the PEA by a digital-to-analog converter and amplified by a voltage amplifier later on. The displacement output of the PEA was detected by a position sensor and sampled by an analog-to-digital converter later on. The experiments were implemented by using the host computer with the MATLAB/Simulink software installed.

### 3.1. Tracking of Sinusoidal Trajectories with Diverse Frequencies

**Figure 4:**
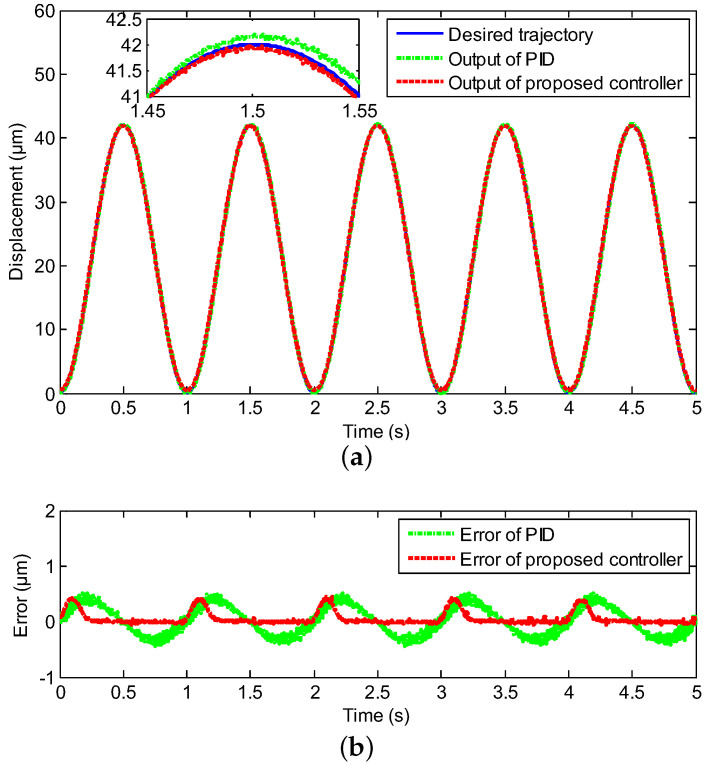
Compared tracking performance of the PEA under the 1 Hz sinusoidal reference, including (**a**) the controller output tracking curve and (**b**) control error.

**Figure 5:**
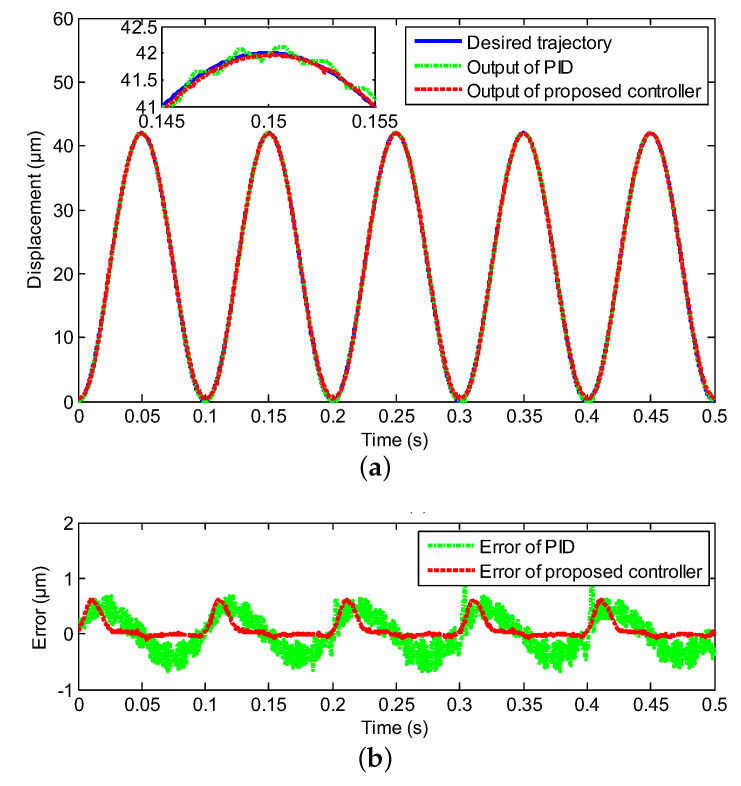
Compared tracking performance of the PEA under the 10 Hz sinusoidal reference, including (**a**) the controller output tracking curve and (**b**) control error.

**Figure 6:**
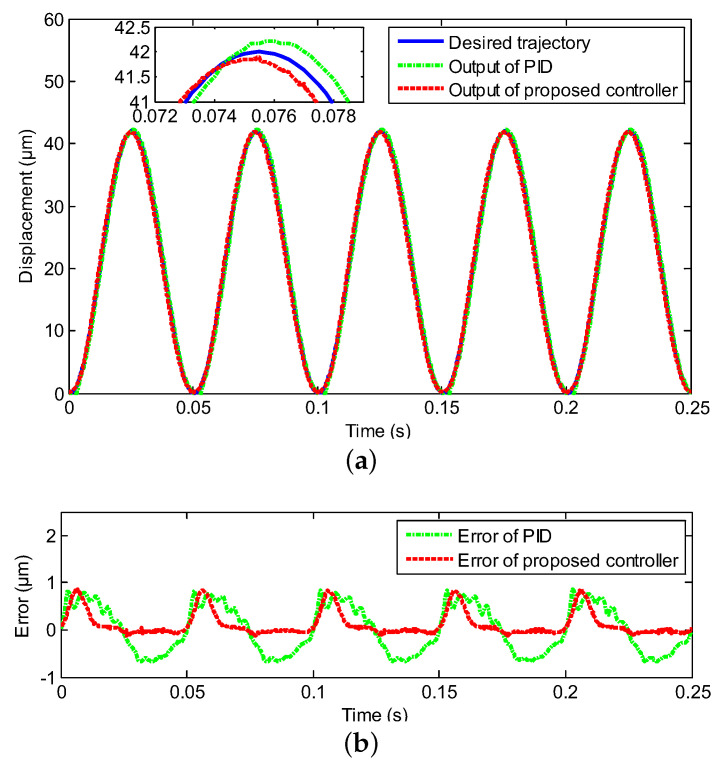
Compared tracking performance of the PEA under the 20 Hz sinusoidal reference, including (**a**) the controller output tracking curve and (**b**) control error.

**Figure 7:**
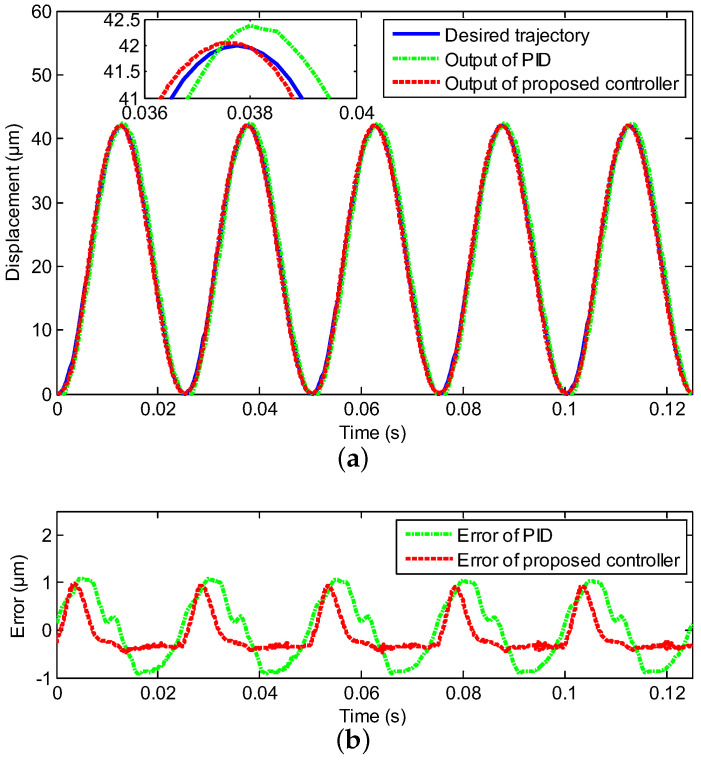
Compared tracking performance of the PEA under the 40 Hz sinusoidal reference, including (**a**) the controller output tracking curve and (**b**) control error.

In this section, the experimental test is deployed to follow the sinusoidal signal under the various frequencies with the proposed neural network for self-tuning control approach, where the reference trajectories were $y\left(t\right)=21+21\sin\left(2\pi \omega t-\frac{\pi }{2}\right)$, ω=1,10,20,40, respectively, and the classical PID control was utilized as a comparison. The compared experimental results, including controller output tracking curves and control errors, are clearly depicted in Figure 4, Figure 5, Figure 6 and Figure 7, where excellent tracking accuracies of the proposed controller are demonstrated.

**Figure 8:**
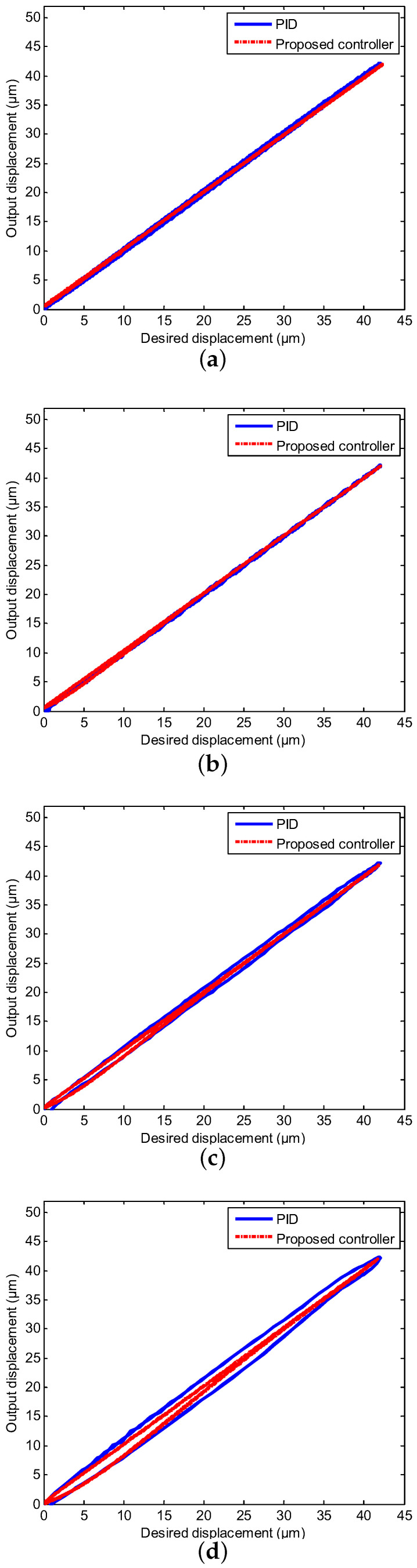
Compared input-output relations of the neural network self-tuning controller and PID controller under the sinusoidal trajectories with diverse input frequencies. (**a**) 1 Hz; (**b**) 10 Hz; (**c**) 20 Hz; (**d**) 40 Hz.

The real displacement outputs of the PEA are marked as the red line, and it can track the desired trajectories noted by the blue line effectively. The errors caused by the model uncertainties and hysteresis nonlinearity could be decreased owing to the proposed neural network self-tuning control approach. Combined with the above advantage, the trajectory tracking errors decreased to a satisfying range. Furthermore, the tracking errors were evaluated by two types of performance indicators, i.e., the root mean squared error (RMSE) and the maximum error (MAXE) were calculated to assess the experimental results. As shown in Table 2, the MAXEs of the proposed controller under the different input frequencies were 1.02%, 1.26%, 1.85%, and 2.45%, respectively, and they were 50%, 55%, 26%, and 25% less than those of the classical PID controller. In addition, the RMSEs were 0.1133 μm, 0.2431 μm, 0.2545 μm, and 0.3148 μm with diverse input frequencies of 1 Hz, 10 Hz, 20 Hz, and 40 Hz by the proposed neural network self-tuning control approach, and they were reduced by 49%, 22 %, 47%, and 40%, respectively, compared with the classical PID controller. The input-output relations of the neural network self-tuning control system are shown in Figure 8, which illustrates that the proposed scheme was valid in diverse frequencies. Furthermore, compared with the PID control method, the hysteresis nonlinearity of the PEA could be significantly suppressed by the proposed controller, and the high-precision tracking goal was achieved via our developed control approach. In addition, in spite of the tracking errors arising by the rate-dependent hysteresis nonlinearity being increased with the growth of frequency, the tracking errors were still within the allowable range. Moreover, according to Table 1, the nominal displacement of the PEA was (50 ± 10%) μm, and the maximum displacement in the experimental results was 42 μm. This implied that our experimental range covered (84.84 ± 8.48)% of the nominal displacement, which demonstrated the rationality of our experiments for eliminating the hysteresis within this range. Therefore, it was obvious that excellent tracking accuracies of the proposed controller could be obtained, which demonstrated the effectiveness of the developed control method.

### 3.2. Tracking of Mixed Triangular Trajectory

**Figure 9:**
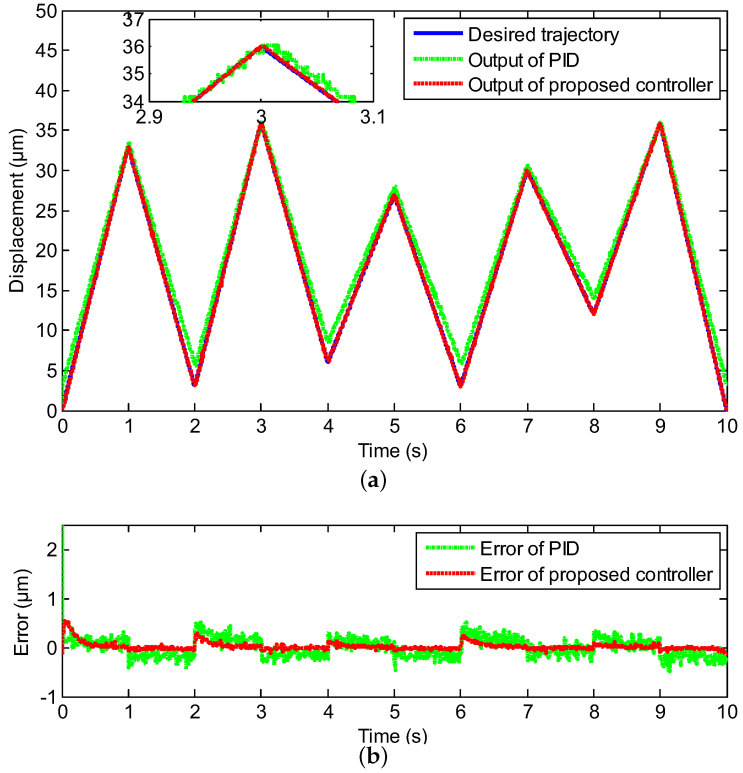
Compared tracking performance of the PEA under the mixed triangular reference, including (**a**) the controller output tracking curve and (**b**) control error.

**Figure 10:**
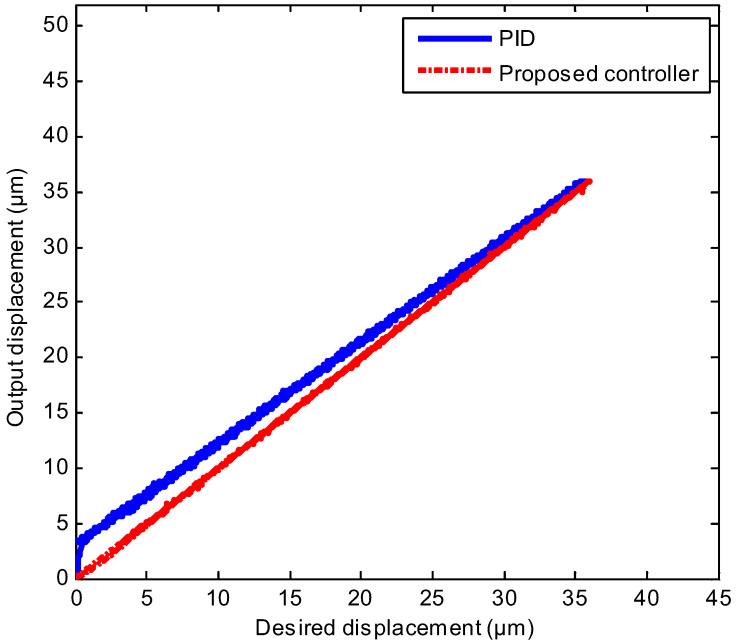
Compared input-output relations of the neural network self-tuning controller and PID controller with the mixed triangular reference.

To further verify the excellent performance of the proposed method, a more complex trajectory tracking experiment was executed, where the desired trajectory was the mixed triangular wave with different amplitudes. The mixed triangular trajectory tracking result is depicted in Figure 9, where the real displacement outputs of the proposed controller marked with the red line could track the desired trajectory marked with the blue line well. Figure 10 represents the input-output relation of the neural network self-tuning controller and the PID controller. As shown in Figure 10, compared with the PID controller, the output displacement and the desired displacement could almost maintain a linear relationship, which manifested the validity of the proposed scheme when tracking the mixed triangular reference. The MAXE and RMSE of constructed controller were 1.56% and 0.1245 μm, and compared with the PID controller, they were improved by 54% and 47%, respectively, which demonstrated that the neural network self-tuning control scheme was effective for the complex trajectory tracking requirement.

In the cases of sinusoidal reference trajectories with various frequencies and the mixed triangular reference trajectory with different amplitudes, the MAXEs and RMSEs were less than 2.45% and 0.3148 μm, respectively. The performance indicators revealed that the constructed control strategy had a satisfactory tracking performance in terms of the different reference trajectories.

## 4. Conclusions

In this paper, a neural network self-tuning control approach described as a nonlinear equation with two unknown variables was proposed to enhance the displacement tracking performance of the PEA. Using the prominent approximation capability of the neural networks, two unknown variables were automatically updated by the neural network identifiers without off-line identification, and then, the neural network self-tuning controller was constructed. The extremely distinguishable characteristic of the proposed strategy was that the hysteresis model was no longer a requisite, which avoided the complex process of the hysteresis modeling and the imprecise inversion issue occurred in inversion based control methods. In addition, the developed controller had the adaptive ability to adjust the system unknown functions owing to the neural network identifiers. Finally, the proposed control methodology was successfully implemented on a commercial PEA product, the comparative experimental results of which indicated that the neural network self-tuning controller had excellent performance in trajectory tracking.

## Figures and Tables

**Table 1:** Basic properties of the PEA product.

Basic Properties	Values	Units
Dimension	7 × 7 × 42	mm
Nominal displacement	50 ± 10%	μm
Maximum push force	1800	N
Stiffness	36	N/μm
Capacitance	6.9 ± 20%	μF

**Table 2:** Compared tracking errors under the sinusoidal trajectories with diverse input frequencies.

Input	PID	Proposed Controller
Frequencies	(RMSE (μm)/MAXE (%))	(RMSE (μm)/MAXE (%))
1 Hz	0.2223/2.04	0.1133/1.02
10 Hz	0.3136/2.77	0.2431/1.26
20 Hz	0.4788/2.51	0.2545/1.85
40 Hz	0.5283/3.29	0.3148/2.45
